# Potential factors for and the prognostic impact of ascites after allogeneic hematopoietic stem cell transplantation

**DOI:** 10.1038/s41598-023-39604-6

**Published:** 2023-08-10

**Authors:** Hiroyuki Kubo, Osamu Imataki, Tetsuya Fukumoto, Yui Kawanaka, Tomoya Ishida, Yukiko Hamasaki Kubo, Jun-ichiro Kida, Makiko Uemura, Haruyuki Fujita, Norimitsu Kadowaki

**Affiliations:** 1 Division of Hematology, Department of Internal Medicine, Faculty of Medicine, Kagawa University, 1750-1 Ikenobe, Miki-Town, Kita-County, Kagawa 761-0793 Japan; 2 Department of Hematology, Takamatsu Red Cross Hospital, Takamatsu, Kagawa Japan; 3 Department of Hematology, Kagawa Prefectural Central Hospital, Takamatsu, Kagawa Japan

**Keywords:** Haematological cancer, Haematological cancer, Risk factors, Comorbidities

## Abstract

Ascites is sometimes detected after allogeneic hematopoietic stem cell transplantation (allo-HSCT); however, since limited information is currently available, its clinical meaning remains unclear. Therefore, we herein examined potential factors for and the impact of ascites on the prognosis of patients after allo-HSCT at our institutes. Fifty-eight patients developed ascites within 90 days of allo-HSCT (small in 34 (16%), moderate-large in 24 (11%)). A multivariate analysis identified veno-occlusive disease/sinusoidal obstruction syndrome (*p* = 0.01) and myeloablative conditioning (*p* = 0.01) as significant potential factors for the development of small ascites. Thrombotic microangiopathy (TMA) (*p* < 0.01) was a significant potential factor for moderate-large ascites. The incidence of both small and moderate-large ascites correlated with lower overall survival (*p* = 0.03 for small ascites and *p* < 0.01 for moderate-large ascites) and higher non-relapse mortality rates (*p* = 0.03 for small ascites and *p* < 0.01 for moderate-large ascites). Lower OS and higher NRM rates correlated with the incidence of both small and moderate-large ascites. Further investigation is warranted to establish whether the clinical sign of ascites improves the diagnostic quality of TMA in a large-scale study.

## Introduction

Refractory and relapsed hematological malignancies generally have a poor prognosis. Allogeneic hematopoietic stem cell transplantation (allo-HSCT) is a well-established curative approach for these diseases. Conditioning regimens before allo-HSCT eliminate residual malignant cells, and allo-HSCT, which involves the replacement of a recipient’s bone marrow with donor hematopoietic stem cells, induces the graft-versus-leukemia/lymphoma effect to minimal residual disease. Although allo-HSCT achieves the highest rate of a complete recovery, the non-relapse mortality (NRM) rate remains high. The non-infectious complications of allo-HSCT include graft-versus-host disease (GVHD), thrombotic microangiopathy (TMA), veno-occlusive disease/sinusoidal obstruction syndrome (VOD/SOS), and organ failure caused by conditioning chemotherapy and GVHD prophylaxis. Ascites is sometimes detected after allo-HSCT. Representative complications showing the clinical sign of ascites after allo-HSCT are VOD/SOS and acute or chronic GVHD^1,2^. The clinical sign of ascites has been incorporated into the diagnostic criteria for VOD/SOS^3–5^. However, we frequently encounter cases of ascites without these complications. Limited information is currently available on the development of ascites after allo-HSCT, including its frequency, potential factors, and impact on the prognosis of patients.

Therefore, a double-center collaborative retrospective study was herein performed on the development of ascites after allo-HSCT in 210 patients at our institutes to investigate its frequency, potential factors, and impact on patient prognosis.

## Methods

### Patients

Between 2000 and 2021, 260 consecutive patients were retrospectively recruited after first allo-HSCT at our institutes (Faculty of Medicine, Kagawa University and Takamatsu Red Cross Hospital, Kagawa, Japan). Fifty patients were excluded due to a lack of data on ascites (48/260 patients, 18%) and primary disease (2/260, 0.8%; multiple myeloma). Therefore, 210 patients were ultimately examined. The Institutional Review Boards of the Faculty of Medicine, Kagawa University and Takamatsu Red Cross Hospital approved the present study.

Abdominal computed tomography (CT) and abdominal ultrasound findings after allo-HSCT were reviewed. These imaging modalities were employed to identify the cause of clinical symptoms, including infections, elevated C-reactive protein levels, and organ dysfunction. Eligible patients were those in whom ascites was detected by a radiologist or abdominal ultrasound technician and confirmed by more than two physician researchers. In the present study, ascites was stratified into small and moderate-large, and defined as that in the rectovesical pouch and out of the rectovesical pouch by CT or abdominal ultrasound, respectively. Ascites limited to the rectovesical pouch was defined as small ascites, while that both in and out of the rectovesical pouch or only out of the rectovesical pouch was defined as moderate-large ascites.

### Transplantation procedures

The classification of conditioning regimens by the Reduced-Intensity Conditioning Regimen workshop by the Center for International Blood and Marrow Transplant Research as myeloablative (MAC) or reduced intensity (RIC) was used in the present study^6,7^. GVHD prophylaxis was performed by the administration of the calcineurin inhibitors (CNI), cyclosporine and tacrolimus alone or in combination with mycophenolate mofetil (MMF) or short-term methotrexate (stMTX, 10-7-7 or 10-7-7-7 mg/m^2^). Anti-thymocyte globulin (ATG) was also administered to a subset of patients.

### Definitions

The previously reported definition for the refined disease risk index (DRI) was employed in the present study^8^. An absolute neutrophil count of 0.5 × 10^9^/L or higher on three consecutive days confirmed engraftment. Acute GVHD was diagnosed and graded according to the modified Glucksberg-Seattle criteria^9,10^. VOD/SOS and TMA were diagnosed according to the Seattle^3^, Baltimore^4^, EBMT^5^, BMT-CTN^11^, or IWG-EBMT criteria^12^. The number of days from transplantation to the confirmation of infection was used as the onset day of viral infection. The detection of 3 cytomegalovirus (CMV)-positive cells per 2 slides using the C10/C11 antigenemia assay or 2 CMV-positive cells per 50,000 white blood cells using the HRP-C7 antigenemia assay confirmed the reactivation of CMV.

### Statistical analysis

In the present study, the 3-year overall survival (OS) rate was used as the primary endpoint, while 3-year cumulative incidence of relapse (CIR) and NRM rates and the relationships between patient characteristics and ascites after allo-HSCT were the secondary endpoints. OS was defined as the time from the landmark point (described below) until death from any cause. Morphological and/or imaging findings were used to diagnose disease relapse/progression. CIR and NRM were defined as the first documented relapse or progression and death without evidence of relapse or progression, respectively. The Kaplan–Meier method was employed to estimate OS probabilities, which were then compared using the Log-rank test. CIR and NRM probabilities were examined using cumulative incidence curves adjusted for competing risks and then compared with Gray’s method. The Cox proportional hazard regression model was employed to identify the factors influencing OS, and the Fine-Gray competing risk regression model for CIR and NRM. Univariate and multivariate analyses were performed to identify potential factors for ascites after allo-HSCT.

Regarding each potential factor, the univariate analysis compared the frequency of categorical or continuous variables between patients with and without ascites. Variables with a *P* value < 0.1 in the univariate analysis were then examined in the multivariate analysis and included age at allo-HSCT (younger than 50 vs. 50 years or older), sex (male vs. female), serum albumin levels before allo-HSCT (continuous variable), the primary disease (myeloid neoplasms vs. lymphoid and ambiguous lineage neoplasms), donor type (human leukocyte antigen [HLA]-matched related donor [MRD]/HLA-matched unrelated donor [MUD] vs. HLA-mismatched related donor [MMRD]/HLA-mismatched unrelated donor [MMUD] vs. cord blood [CB] vs. haploidentical), the use of ATG, the type of GVHD prophylaxis (CNI plus MTX vs. others), the type of conditioning regimen (MAC vs. RIC), the hematopoietic cell transplant-comorbidity index (0 vs. ≥ 1), neutrophil engraftment (< day 18 vs. ≥ day 18), and the occurrence of G2-4 acute GVHD (No vs. Yes), VOD/SOS (No vs. Yes), TMA (No vs. Yes), CMV viremia (No vs. Yes), and disease recurrence (No vs. Yes). The effects of neutrophil engraftment, G2-4 acute GVHD, VOD/SOS, TMA, CMV viremia, and disease recurrence on the development of ascites and clinical outcomes were investigated in patients who survived for 90 days after allo-HSCT. This landmark method excluded any potential bias caused by the inclusion of patients who died before these clinical events manifested in the group without these clinical events^13,14^.

Statistical analyses were all two-sided and *P*-values < 0.05 indicated a significant difference. Statistical analyses were conducted using EZR (version 1.33) (Saitama Medical Center, Jichi Medical University)^15^.

### Ethical approval

The study complied with the declaration of Helsinki. This study was approved by the Institutional Review Boards of the Faculty of Medicine, Kagawa University and Takamatsu Red Cross Hospital.

### Consent to participate

Informed consent was obtained from all individual participants included in the study.

## Results

### Patient characteristics

Table 1 shows the characteristics of patients who were retrospectively recruited in the present study. The median age of patients at allo-HSCT was 52 years (range, 16–74), and 113 (54%) were 50 years or older. There were 119 male patients (57%). The median serum albumin level before allo-HSCT was 4.0 g/dL (range, 2.7–5.1). Primary diseases were as follows: myeloid neoplasms in 128 (61%: acute myeloid leukemia in 84, myelodysplastic syndrome in 35, myeloproliferative neoplasms in 4, and chronic myelogenous leukemia in 5) and lymphoid and ambiguous lineage neoplasms in 82 (39%: precursor or ambiguous lineage neoplasms in 53 and mature neoplasms in 29). Refined DRI were low, intermediate, high, and very high in 16 (8%), 85 (40%), 72 (34%), and 37 (18%) patients, respectively. Donor types were MRD, MMRD, MUD, MMUD, CB, and haploidentical in 39 (19%), 5 (2%), 77 (37%), 56 (27%), 28 (13%), and 5 (2%) patients, respectively. GVHD prophylaxis involved CNI plus MTX in 182 patients (87%) and others in 28 (13%), while ATG was administered to a subset of 53 patients (25%). Regarding conditioning regimens, 147 patients received MAC (70%), while 63 were treated with RIC (30%). The median follow-up period among surviving patients was 44 months (range, 1–209).

**Table 1:** Characteristics of patients with ascites within 90 days of allo-HSCT.

**Characteristics**	**Total**
	**n (%)**
**Age**	
< 50	97 (46.2)
≥ 50	113 (53.8)
**Sex**	
Male	119 (56.7)
Female	91 (43.3)
**Primary disease**	
Myeloid neoplasms	128 (61.0)
Lymphoid and ambiguous lineage neoplasms	82 (39.0)
**Refined disease risk index**	
Low/intermediate	101 (48.1)
High/very high	109 (51.9)
**Donor source**	
MRD/MUD	116 (55.2)
MMRD/MMUD	61 (29.0)
CB	28 (13.3)
Haploidentical	5 (2.4)
**ATG**	
No	157 (74.8)
Yes	53 (25.2)
**GVHD prophylaxis**	
CNI + MTX	182 (86.7)
Others	28 (13.3)
**Conditioning regimen**	
MAC	147 (70.0)
RIC	63 (30.0)
**HCT-CI Total Points**	
0	157 (74.8)
≥ 1	53 (25.2)
**Neutrophil engraftment**	
< day 18	127 (60.5)
≥ day 18	79 (37.6)
No data	4 (1.9)
**G2-4 acute GVHD within 90 days**	
No	132 (62.9)
Yes	78 (37.1)
**VOD/SOS**	
No	188 (89.5)
Yes	22 (10.5)
**TMA within 90 days**	
No	191 (91.0)
Yes	19 (9.0)
**CMV viremia within 90 days**	
No	146 (69.5)
Yes	64 (30.5)
**Disease recurrence within 90 days**	
No	187 (89.0)
Yes	23 (11.0)
**Ascites within 90 days**	
No	152 (72.4)
Small	34 (16.2)
Moderate-large	24 (11.4)
**Ascites before allo-HSCT among 58 patients with ascites**	
No	45 (77.6)
Small	4 (6.9)
Moderate-large	1 (1.7)
No data	8 (13.8)

*MRD* HLA-matched related donor, *MUD* HLA-matched unrelated donor, *MMRD* HLA-mismatched related donor, *MMUD* HLA-mismatched unrelated donor, *CB* cord blood, *ATG* anti-thymocyte globulin, *GVHD* graft-versus-host disease, *CNI* calcineurin inhibitor, *MTX* methotrexate, *MAC* myeloablative conditioning, *RIC* reduced intensity conditioning, *HCT-CI* hematopoietic cell transplant-comorbidity index, *VOD/SOS* veno-occlusive disease/sinusoidal obstruction syndrome, *TMA* thrombotic microangiopathy, *CMV* cytomegalovirus, *allo-HSCT* allogeneic hematopoietic stem cell transplantation.

### Potential factors for ascites

Ascites was detected in 58 patients within 90 days of allo-HSCT (small in 34 (16%), moderate-large in 24 (11%)). None of the patients in the present cohort underwent surgical interventions. The univariate analysis identified late neutrophil engraftment (HR, 2.65; 95% CI 1.13–6.20; *p* = 0.03), G2-4 acute GVHD (HR, 2.36; 95% CI 1.03–5.39; *p* = 0.04), and VOD/SOS (HR, 5.82; 95% CI 1.72–19.70; *p* < 0.01) as significant potential factors for small ascites. RIC (HR, 0.27; 95% CI 0.08–0.95; *p* = 0.04) was also identified as a potential factor (Table 2). In the multivariate analysis, VOD/SOS (HR, 7.19; 95% CI 1.52–34.00; *p* = 0.01) and RIC (HR, 0.13; 95% CI 0.03–0.66; *p* = 0.01) remained as significant potential factors associated with small ascites.

**Table 2:** Univariate and multivariate analyses of potential factors for small ascites within 90 days of allo-HSCT.

Variable	n	Univariate: Logistic regression		Multivariate: Logistic regression	
		Odds ratio (95% CI)	*P* value	Odds ratio (95% CI)	*P* value
Age					
< 50	97	1	0.63	–	–
≥ 50	113	0.82 (0.36–1.85)		–	
Sex					
Male	119	1	0.11	–	–
Female	91	1.98 (0.87–4.50)		–	
Primary disease					
Myeloid neoplasms	128	1	0.22	–	–
Lymphoid and ambiguous lineage neoplasms	82	0.57 (0.24–1.40)		–	
Refined disease risk index					
Low/intermediate	101	1	0.77	–	–
High/very high	109	1.13 (0.50–2.55)		–	
Donor source					
MRD/MUD	116	1		–	
MMRD/MMUD	61	0.51 (0.18–1.46)	0.21	–	–
CB	28	1.04 (0.27–4.05)	0.96	–	–
Haploidentical	5	2.08 (0.18–24.10)	0.56	–	–
ATG					
No	157	1	0.90	–	–
Yes	53	1.06 (0.41–2.73)		–	
GVHD prophylaxis					
CNI + MTX	182	1	0.97	–	–
Others	28	1.03 (0.28–3.85)		–	
Conditioning regimen					
MAC	147	1	**0.04**	1	**0.01**
RIC	63	0.27 (0.08–0.95)		0.13 (0.03–0.66)	
HCT-CI Total Points					
0	157	1	0.20	–	–
≥ 1	53	1.77 (0.74–4.22)		–	
Neutrophil engraftment					
< day 18	127	1	**0.03**	1	0.10
≥ day 18	79	2.65 (1.13–6.20)		2.22 (0.85–5.78)	
G2-4 acute GVHD within 90 days					
No	132	1	**0.04**	1	0.05
Yes	78	2.36 (1.03–5.39)		2.44 (0.98–6.09)	
VOD/SOS					
No	188	1	**< 0.01**	1	**0.01**
Yes	22	5.82 (1.72–19.70)		7.19 (1.52–34.00)	
TMA within 90 days					
No	191	1	0.20	–	**–**
Yes	19	3.36 (0.54–21.10)		–	
CMV viremia within 90 days					
No	146	1	0.93	–	–
Yes	64	1.04 (0.43–2.48)		–	
Disease recurrence within 90 days					
No	187	1	0.28	–	–
Yes	23	2.18 (0.53–9.00)		–	

*MRD* HLA-matched related donor, *MUD* HLA-matched unrelated donor, *MMRD* HLA-mismatched related donor, *MMUD* HLA-mismatched unrelated donor, *CB* cord blood, *ATG* anti-thymocyte globulin, *GVHD* graft-versus-host disease, *CNI* calcineurin inhibitor, *MTX* methotrexate, *MAC* myeloablative conditioning, *RIC* reduced intensity conditioning, *HCT-CI* hematopoietic cell transplant-comorbidity index, *VOD/SOS* veno-occlusive disease/sinusoidal obstruction syndrome, *TMA* thrombotic microangiopathy, *CMV* cytomegalovirus, *CI* confidence interval.

Significant values are in bold.

We then investigated the development of moderate-large ascites using the same analyses. The univariate analysis identified TMA (HR, 27.50; 95% CI 7.12–106.00; *p* < 0.01) as a significant potential factor (Table 3), and this factor remained significant in the multivariate analysis (HR, 25.30; 95% CI 6.13–104.00; *p* < 0.01).

**Table 3:** Univariate and multivariate analyses of potential factors for moderate-large ascites within 90 days of allo-HSCT.

Variable	n	Univariate: Logistic regression		Multivariate: Logistic regression	
		Odds ratio (95% CI)	*P* value	Odds ratio (95% CI)	*P* value
Age					
< 50	97	1	0.77	–	–
≥ 50	113	0.85 (0.30–2.46)		–	
Sex					
Male	119	1	0.81	–	–
Female	91	0.88 (0.30–2.58)		–	
Primary disease					
Myeloid neoplasms	128	1	0.28	–	–
Lymphoid and ambiguous lineage neoplasms	82	1.80 (0.62–5.20)		–	
Refined disease risk index					
Low/intermediate	101	1	0.36	–	–
High/very high	109	1.66 (0.56–4.87)		–	
Donor source					
MRD/MUD	116	1		–	
MMRD/MMUD	61	2.13 (0.65–6.97)	0.21	–	–
CB	28	2.18 (0.40–11.80)	0.37	–	–
Haploidentical	5	5.44 (0.49–60.50)	0.17	–	–
ATG					
No	157	1	0.43	–	–
Yes	53	1.58 (0.51–4.89)		–	
GVHD prophylaxis					
CNI + MTX	182	1	**0.08**	1	0.08
Others	28	3.10 (0.89–10.80)		3.69 (0.85–16.00)	
Conditioning regimen					
MAC	147	1	0.55	–	**–**
RIC	63	0.67 (0.18–2.49)		–	
HCT-CI total points					
0	157	1	0.95	–	–
≥ 1	53	1.04 (0.31–3.44)		–	
Neutrophil engraftment					
< day 18	127	1	0.18	–	**–**
≥ day 18	79	2.07 (0.71–6.03)		–	
G2-4 acute GVHD within 90 days					
No	132	1	**0.08**	1	0.33
Yes	78	2.62 (0.89–7.72)		1.86 (0.53–6.47)	
VOD/SOS					
No	188	1	0.42	–	**–**
Yes	22	1.92 (0.39–9.53)		–	
TMA within 90 days					
No	191	1	**< 0.01**	1	**< 0.01**
Yes	19	27.50 (7.12–106.00)		25.30 (6.13–104.00)	
CMV viremia within 90 days					
No	146	1	0.50	–	–
Yes	64	1.45 (0.49–4.29)		–	
Disease recurrence within 90 days					
No	187	1	0.99	–	–
Yes	23	0.00 (0.00-Inf)		–	

*MRD* HLA-matched related donor, *MUD* HLA-matched unrelated donor, *MMRD* HLA-mismatched related donor, *MMUD* HLA-mismatched unrelated donor, *CB* cord blood, *ATG* anti-thymocyte globulin, *GVHD* graft-versus-host disease, *CNI* calcineurin inhibitor, *MTX* methotrexate, *MAC* myeloablative conditioning, *RIC* reduced intensity conditioning, *HCT-CI* hematopoietic cell transplant-comorbidity index, *VOD/SOS* veno-occlusive disease/sinusoidal obstruction syndrome, *TMA* thrombotic microangiopathy, *CMV* cytomegalovirus, *CI* confidence interval.

Significant values are in bold.

Serum albumin levels were not associated with the occurrence of small (*p* = 0.34) or moderate-large (*p* = 0.16) ascites in the univariate analysis.

Clinical diagnoses of ascites (n = 58) designated by the primary physician in the present cohort included infection in 6 patients (10%: small ascites in 5, moderate-large ascites in 1), GVHD in 17 (29%: small in 13, moderate-large in 4), VOD/SOS in 6 (10%: small in 4, moderate-large in 2), TMA in 12 (21%: small in 3, moderate-large in 9), and others in 17 (29%: small in 9, moderate-large in 8). Infections were treated by antibiotics (n = 6), GVHD by increasing immunosuppression using corticosteroids (n = 15) or MMF (n = 2), VOD/SOS using fresh frozen plasma (n = 6), and TMA by dose reductions in or discontinuing CNI (n = 10), fresh frozen plasma (n = 5), and recombinant thrombomodulin (n = 3) (the total number of treatment procedures). In cases in which it was possible to assess treatment effects, response rates for ascites were 75% (3 out of 4 patients) in infection cases, 73% (8 out of 11) in GVHD cases, 60% (3 out of 5) in VOD/SOS cases, and 75% (6 out of 8) in TMA cases. Furthermore, the attenuation of ascites was noted in 6 out of 8 patients with a good clinical response to the TMA treatment, in contrast to none of the patients with a poor clinical response.

### Transplant outcomes

#### OS, CIR, and NRM

**Figure 1:**
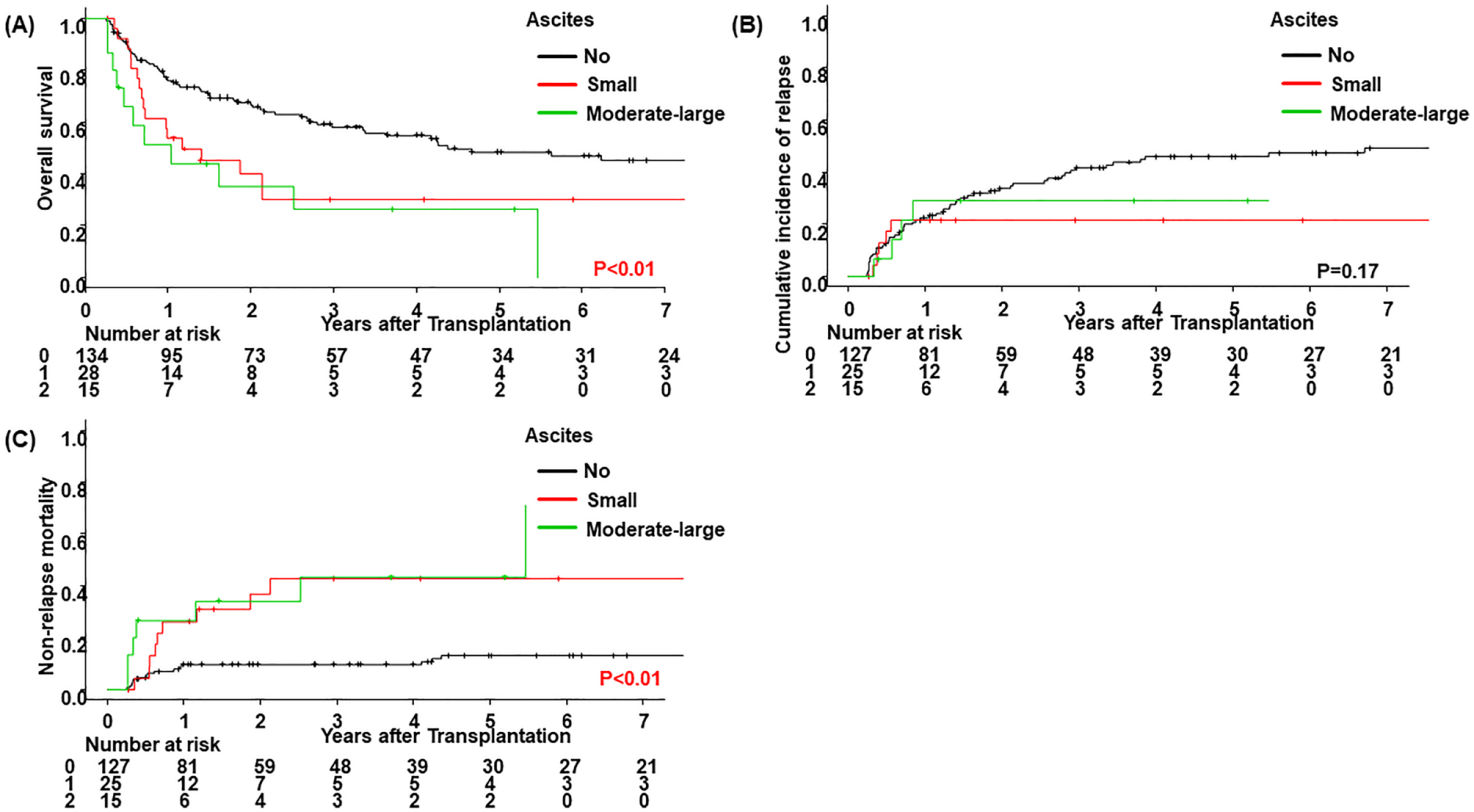
Clinical outcomes by the status of ascites within 90 days of allo-HSCT. (**A**) Overall survival, (**B**) cumulative incidence of relapse, and (**C**) non-relapse mortality were stratified according to the status of ascites within 90 days of allo-HSCT (no vs. small vs. moderate-large).

**Figure 2:**
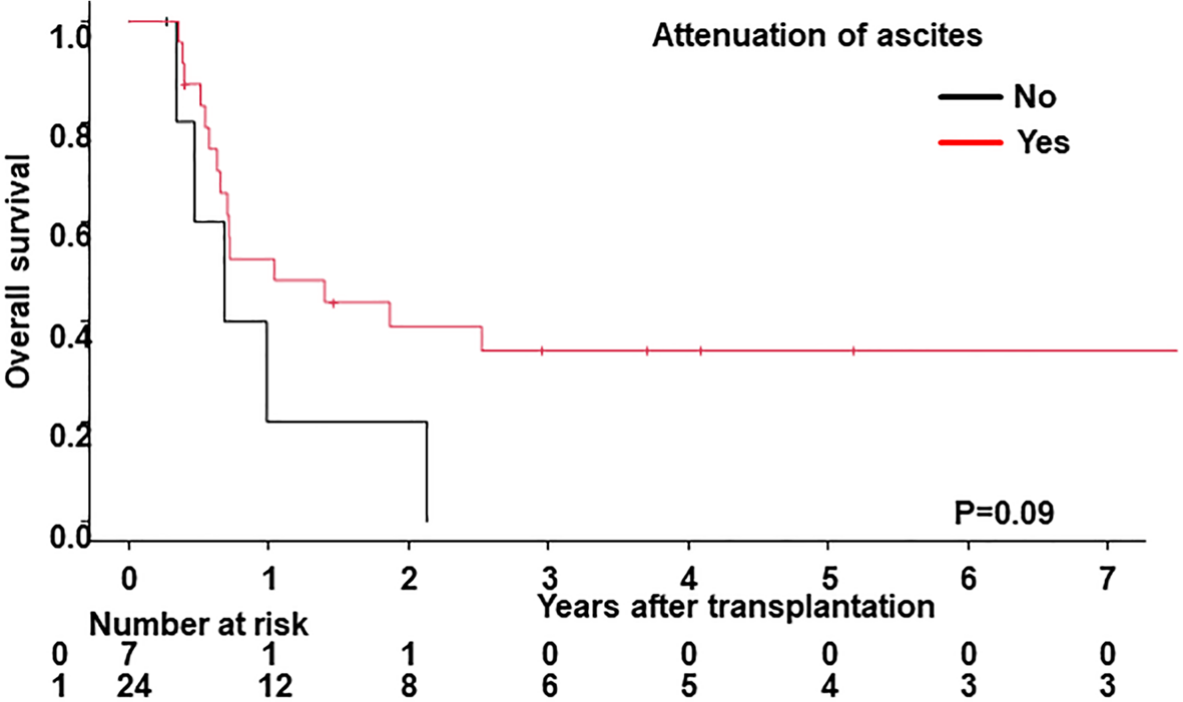
Clinical outcomes according to responses to treatment for ascites within 90 days of allo-HSCT. Overall survival was stratified according to responses to treatment for ascites within 90 days of allo-HSCT (no vs. yes).

Three-year OS, CIR, and NRM rates in the present cohort were 51% (95% CI 43–59), 38% (95% CI 30–46), and 17% (95% CI 12–24), respectively. Following the stratification of ascites 90 days after allo-HSCT (no vs. small vs. moderate-large), a significantly lower OS rate was noted in patients with small (*p* = 0.03) and moderate-large ascites (*p* < 0.01). No significant differences were observed in the CIR rate in patients with small ascites (*p* = 0.10), whereas a significantly higher NRM rate was noted in patients with small (*p* < 0.01) and moderate-large ascites (*p* < 0.01) (Fig. 1, Table 4). In the multivariate analysis, the incidence of both small and moderate-large ascites correlated with a lower OS rate (HR, 1.85; 95% CI 1.06–3.21; *p* = 0.03 for small ascites and HR, 2.56; 95% CI 1.30–5.04; *p* < 0.01 for moderate-large ascites) (Table 5). The incidence of small ascites did not correlate with a lower CIR rate (HR, 0.37; 95% CI 0.13–1.08; *p* = 0.07). In contrast, correlations were detected between the incidence of both small and moderate-large ascites and a higher NRM rate (HR, 2.80; 95% CI 1.09–7.19; *p* = 0.03 for small ascites and HR, 4.31; 95% CI 1.75–10.63; *p* < 0.01 for moderate-large ascites). Additionally, we compared the OS rate between patients who responded to treatment for ascites and those who did not. In patients with and without clear causes of ascites in whom it was possible to assess treatment effects, the attenuation of ascites was confirmed in 27 out of 38. No significant difference was observed in the OS rate (*p* = 0.09) between these groups; however, it was slightly higher in patients showing the attenuation of ascites (Fig. 2).

**Table 4:** Univariate analysis of risk factors affecting clinical outcomes.

Variable	n	OS		CIR		NRM	
		Univariate					
		Hazard ratio (95% CI)	*P* value	Hazard ratio (95% CI)	*P* value	Hazard ratio (95% CI)	*P* value
Age							
< 50	97	1	0.19	1	0.76	1	0.47
≥ 50	113	1.32 (0.87–1.99)		1.08 (0.67–1.74)		1.29 (0.65–2.56)	
Sex							
Male	119	1	**< 0.01**	1	0.88	1	**0.04**
Female	91	1.76 (1.16–2.66)		1.04 (0.63–1.70)		2.06 (1.03–4.12)	
Primary disease							
Myeloid neoplasms	128	1	0.70	1	0.12	1	0.76
Lymphoid and ambiguous lineage neoplasms	82	0.92 (0.60–1.41)		0.67 (0.40–1.11)		1.12 (0.55–2.25)	
Refined disease risk index							
Low/intermediate	101	1	**< 0.01**	1	**< 0.01**	1	0.34
High/very high	109	2.04 (1.34–3.12)		2.49 (1.53–4.04)		0.71 (0.35–1.44)	
Donor source							
MRD/MUD	116	1		1		1	
MMRD/MMUD	61	0.61 (0.36–1.03)	**0.06**	0.36 (0.18–0.72)	**< 0.01**	1.16 (0.54–2.49)	0.71
CB	28	1.75 (0.94–3.27)	**0.08**	1.74 (0.90–3.39)	**0.10**	1.11 (0.33–3.80)	0.87
Haploidentical	5	3.35 (1.03–10.86)	**0.04**	0.80 (0.12–5.35)	0.82	2.46 (0.29–20.95)	0.41
ATG							
No	157	1	**0.08**	1	0.71	1	0.20
Yes	53	0.63 (0.37–1.06)		0.90 (0.51–1.59)		0.54 (0.21–1.39)	
GVHD prophylaxis							
CNI + MTX	182	1	0.18	1	0.17	1	0.73
Others	28	1.47 (0.83–2.61)		1.57 (0.82–3.00)		0.81 (0.24–2.69)	
Conditioning regimen							
MAC	147	1	0.92	1	0.87	1	0.96
RIC	63	0.98 (0.61–1.56)		0.96 (0.57–1.62)		0.98 (0.44–2.17)	
G2-4 acute GVHD within 90 days							
No	132	1	0.55	1	**0.02**	1	**0.01**
Yes	78	1.14 (0.74–1.75)		0.53 (0.31–0.92)		2.38 (1.20–4.75)	
Ascites within 90 days							
No	152	1		1		1	
Small	34	1.86 (1.08–3.19)	**0.03**	0.45 (0.17–1.18)	**0.10**	3.64 (1.64–8.11)	**< 0.01**
Moderate-large	24	2.66 (1.40–5.07)	**< 0.01**	0.59 (0.21–1.69)	0.33	5.11 (2.14–12.23)	**< 0.01**

*MRD* HLA-matched related donor, *MUD* HLA-matched unrelated donor, *MMRD* HLA-mismatched related donor, *MMUD* HLA-mismatched unrelated donor, *CB* cord blood, *ATG* anti-thymocyte globulin, *GVHD* graft-versus-host disease, *CNI* calcineurin inhibitor, *MTX* methotrexate, *MAC* myeloablative conditioning, *RIC* reduced intensity conditioning, *OS* overall survival, *CIR* cumulative incidence of relapse, *NRM* non-relapse mortality, *CI* confidence interval.

Significant values are in bold.

**Table 5:** Multivariate analysis of risk factors affecting clinical outcomes.

Variable	n	OS		CIR		NRM	
		Multivariate					
		Hazard ratio (95% CI)	*P* value	Hazard ratio (95% CI)	*P* value	Hazard ratio (95% CI)	*P* value
Sex							
Female	91	1.78 (1.16–2.71)	**< 0.01**	–	–	–	–
Refined disease risk index							
High/very high	109	1.92 (1.23–3.01)	**< 0.01**	2.51 (1.50–4.19)	**< 0.01**	–	–
Donor source							
MMRD/MMUD	61	–	–	0.34 (0.17–0.66)	**< 0.01**	–	–
ATG							
Yes	53	0.55 (0.32–0.97)	**0.04**	–	–	–	–
Ascites within 90 days							
No	152	1		1		1	
Small	34	1.85 (1.06–3.21)	**0.03**	0.37 (0.13–1.08)	0.07	2.80 (1.09– 7.19)	**0.03**
Moderate-large	24	2.56 (1.30–5.04)	**< 0.01**	0.55 (0.19–1.62)	0.28	4.31 (1.75–10.63)	**< 0.01**

*MMRD* HLA-mismatched related donor, *MMUD* HLA-mismatched unrelated donor, *ATG* anti-thymocyte globulin, *OS* overall survival, *CIR* cumulative incidence of relapse, *NRM* non-relapse mortality, *CI* confidence interval.

Significant values are in bold.

Correlations were also observed between a lower OS rate and being female as well as having a high/very high refined DRI, while a higher OS rate correlated with the use of ATG. A higher CIR rate correlated with a high/very high refined DRI, while a lower CIR rate correlated with MMRD/MMUD. The following causes of NRM (n = 42) were identified: infectious disease in 12 patients (29%), GVHD in 13 (31%), organ failure in 3 (7%), secondary malignancy in 2 (5%), bleeding in 3 (7%), TMA in 5 (12%), engraftment failure in 3 (7%), and suicide in 1 (2%).

Additionally, 41 out of 58 patients with ascites died during the follow-up period. The following causes of death were identified: disease recurrence in 12 patients (29%), infectious disease in 7 (17%), GVHD in 9 (22%), organ failure in 3 (7%), secondary malignancy in 1 (2%), bleeding in 4 (10%), TMA in 3 (7%), engraftment failure in 1 (2%), and suicide in 1 (2%).

## Discussion

Limited information is currently available on the potential factors associated with ascites after allo-HSCT; therefore, a comprehensive analysis of potential factors was performed herein. We previously examined potential factors for and the prognostic impact of pericardial effusion after allo-HSCT^16^. Based on the findings obtained, we herein investigated potential factors for ascites after allo-HSCT. The multivariate analysis identified VOD/SOS and MAC as potential factors for small ascites within 90 days of allo-HSCT. G2-4 acute GVHD was also associated with the development of ascites. G2-4 acute GVHD, VOD/SOS, and MAC were regarded as acceptable potential factors for ascites after allo-HSCT. Previous studies reported that acute GVHD^1^ and VOD/SOS^17,18^ exhibited the clinical sign of ascites for a number of reasons, such as inflammation and sinusoidal endothelial cell injury. Although MAC is associated with intensive donor immune suppression and antitumor effects, cardiac and hepatic toxicities (including VOD/SOS) are caused by cyclophosphamide, total body irradiation, and busulfan included in MAC^19–22^. Organ injury by MAC may show the clinical sign of ascites. In the present study, TMA correlated with moderate-large ascites after allo-HSCT. Furthermore, a previous study reported that TMA induced pericardial and pleural effusion^23,24^. Nevertheless, limited information is currently available on the relationship between TMA and ascites^25^. TMA damages multiple organs, including the heart, kidneys, and gut^23,24^. Organ failure caused by TMA may manifest the clinical sign of ascites. The present results indicate that ascites, particularly moderate-large ascites, reflects the presence of TMA. TMA and VOD/SOS share a common pathogenesis of vascular endothelial syndromes that develop early after HSCT^26^. Vascular endothelial cell damage induces TMA and VOD/SOS, which cause renal, cardiac, and gut or liver damage, ultimately leading to multiple organ failure. Nevertheless, the clinical sign of ascites is not included in the diagnostic criteria of TMA^11,12^, whereas it is one of the diagnostic criteria of VOD/SOS^4,5^. Therefore, ascites may be an important clinical sign in not only VOD/SOS, but also TMA.

A recent study reported a patient who developed transudative refractory ascites secondary to non-cirrhotic, non-VOD/SOS-related portal hypertension after allo-HSCT^27^. Since the NRM rate of this disease entity is 63%, it was concluded to be a previously unrecognized and fatal complication after allo-HSCT. Further studies on the underlying causes of this disease entity are warranted.

Ascites has a negative impact on the NRM rate of allo-HSCT. We investigated the effects of ascites on transplant outcomes. The multivariate analysis revealed correlations between the incidence of both small and moderate-large ascites and poor OS and higher NRM rates. G2-4 acute GVHD was associated with the development of small ascites. Previous studies showed that the rate of CIR was lower in patients with G2-4 acute GVHD^28^. In the present study, small ascites induced by G2-4 acute GVHD was associated with a slightly lower CIR. On the other hand, TMA was the only potential factor for moderate-large ascites. Therefore, moderate-large ascites did not correlate with CIR.

There are a number of limitations that need to be addressed. This was a retrospective analysis that may have had a selection bias. Fifty out of the 260 patients recruited were excluded due to a lack of data on ascites and the small subset group. CT and abdominal ultrasound were conducted on patients with negative clinical signs. Since the times at which these clinical examinations were performed varied, the incidence of ascites may have been underestimated. Furthermore, we excluded patients who died within 90 days of allo-HSCT from the analysis. This study design may also have influenced analysis outcomes. Another limitation is the small number of ascites events, which may have affected the potential factor analysis.

In conclusion, the present results identified VOD/SOS and MAC as significant potential factors for small ascites within 90 days of allo-HSCT. TMA was associated with moderate-large ascites. Lower OS and higher NRM rates correlated with the incidence of both small and moderate-large ascites. The present study is novel because it investigated potential factors for ascites after allo-HSCT as well as the impact of the amount of ascites on the prognosis of patients. A relationship was observed between ascites, particularly moderate-large ascites, and TMA. Further investigation is warranted to establish whether the clinical sign of ascites improves the diagnostic quality of TMA in a large-scale study.

## Data Availability

The data supportive the findings of this study are available on request to the corresponding author. The data are not publicly available due to privacy or ethical restrictions.
